# Antenatal IL-1-dependent inflammation persists postnatally and causes retinal and sub-retinal vasculopathy in progeny

**DOI:** 10.1038/s41598-018-30087-4

**Published:** 2018-08-08

**Authors:** Alexandra Beaudry-Richard, Mathieu Nadeau-Vallée, Élizabeth Prairie, Noémie Maurice, Émilie Heckel, Mohammad Nezhady, Sheetal Pundir, Ankush Madaan, Amarilys Boudreault, Xin Hou, Christiane Quiniou, Estefania Marin Sierra, Alexandre Beaulac, Gregory Lodygensky, Sarah A. Robertson, Jeffrey Keelan, Kristina M. Adams Waldorf, David M. Olson, Jose-Carlos Rivera, William D. Lubell, Jean-Sebastien Joyal, Jean-François Bouchard, Sylvain Chemtob

**Affiliations:** 1 Departments of Pediatrics, Ophthalmology and Pharmacology, CHU Sainte-Justine Research Centre, Montréal, Canada; 2 Department of Pharmacology, Université de Montréal, Montréal, Canada; 3 Department of Pharmacology and Therapeutics, McGill University, Montréal, Canada; 4 Department of Obstetrics and Gynaecology, University of Adelaide, Adelaide, South Australia 5005 Australia; 5 Div Obstetrics & Gynaecology, University of Western Australia King Edward Memorial Hospital, Perth, Australia; 6 Department of Obstetrics & Gynaecology, University of Washington, Seattle, WA USA; 7 Departments of Obstetrics and Gynaecology, Pediatrics and Physiology, University of Alberta, Edmonton, AB Canada; 8 Department of Ophthalmology, Maisonneuve-Rosemont Hospital Research Centre, Montréal, Canada; 9 Department of Chemistry, Université de Montréal, Montréal, Canada; 10 School of Optometry, Université de Montréal, Montréal, Canada

**Keywords:** Target identification, Interleukins, Retinopathy of prematurity, Paediatric research, Translational research

## Abstract

Antenatal inflammation as seen with chorioamnionitis is harmful to foetal/neonatal organ development including to eyes. Although the major pro-inflammatory cytokine IL-1β participates in retinopathy induced by hyperoxia (a predisposing factor to retinopathy of prematurity), the specific role of antenatal IL-1β associated with preterm birth (PTB) in retinal vasculopathy (independent of hyperoxia) is unknown. Using a murine model of PTB induced with IL-1β injection *in utero*, we studied consequent retinal and choroidal vascular development; in this process we evaluated the efficacy of IL-1R antagonists. Eyes of foetuses exposed only to IL-1β displayed high levels of pro-inflammatory genes, and a persistent postnatal infiltration of inflammatory cells. This prolonged inflammatory response was associated with: (1) a marked delay in retinal vessel growth; (2) long-lasting thinning of the choroid; and (3) long-term morphological and functional alterations of the retina. Antenatal administration of IL-1R antagonists – 101.10 (a modulator of IL-1R) more so than Kineret (competitive IL-1R antagonist) – prevented all deleterious effects of inflammation. This study unveils a key role for IL-1β, a major mediator of chorioamnionitis, in causing sustained ocular inflammation and perinatal vascular eye injury, and highlights the efficacy of antenatal 101.10 to suppress deleterious inflammation.

## Introduction

Preterm birth (PTB) still remains a major medical concern^1^. At present, PTB is a primary cause of infant mortality and morbidity in the USA and the rest of the world^2,3^. Globally, more than 10% of infants are born preterm, amounting to 15 million children worldwide^4^. Prematurity disrupts normal organogenesis; this applies to major organs such as lungs, brain, gut, and eyes^5–7^. Exposure to the higher extrauterine O_2_ concentrations (relative to those *in utero*) exerts toxicity to these neonatal organs, including for instance by impairing vessel growth in the eye thus predisposing to the development of retinopathy of prematurity (ROP)^8^.

Other perinatal conditions also contribute to foetal/neonatal organ damage. Along these lines, inflammation (as seen in chorioamnionitis) is reported to contribute to more than 60% of PTB prior to 28 weeks gestation^9,10^. Chorioamnionitis is also deleterious to many foetal/neonatal organs^9,11^, and can lead to foetal inflammatory response syndrome (FIRS). Fragile endothelium is particularly vulnerable and a contributor to vascular dysfunction during acute and chronic inflammation^12,13^, such that FIRS has not only been linked to higher risks of brain injury^14^ but also of that to the retina^15–18^ by contributing to ROP^19^ and associated microvascular degeneration^20^ and retinal dysfunctions^21^. Concurrently, inflammation is known to induce numerous cytotoxic mediators in endothelial cells^22^.

During infection and sterile inflammation, Toll-like receptors are activated by small molecular motifs on bacterial cell walls and/or by molecules released by stressed cells, which in turn induce the expression of pro-inflammatory cytokines and chemokines to further magnify the inflammatory cascade^23^. Inflammatory mediators activate uterine activation proteins, which will favour cervical ripening and foetal membrane weakening, and trigger contractions and labour^23^. Among the major pro-inflammatory cytokines Interleukin (IL)-6 and IL-8 concentrations dominate in the perinatal period^24–26^. However, IL-1β is a trigger and amplifier of the inflammatory cascade, including of IL-6 and IL-8^27,28^. Accordingly, increased levels of IL-1β in the foetal compartment are observed with PTB in humans^29,30^, and polymorphisms of corresponding IL-1 gene pathways affect the risk of preterm birth^31–33^. IL-1β in turn contributes directly to prematurity^34^ and to foetal/neonatal injury^35^. In this context, IL-1β-dependent retinal and sub-retinal injury to the immature subject is clearly described and involves direct endothelial cytotoxicity^36^, but the associated inflammation is mostly secondary to post-natal alterations in oxygen exposure^37–40^. However other than epidemiological links and associated roles in other eye damaging conditions triggered by distinct mechanisms, notably oxidative stress, the direct role for IL-1β in causing injury to the retina/sub-retina of the immature subject is unknown. To discriminate the specific role of antenatal IL-1β (independent of hyperoxia - a predisposing factor to ROP) on oculo-vascular development we studied the effects of gestational IL-1β associated with foetal inflammatory response on development of retinal and choroidal vessels. To ascertain the role of IL-1β we also desirably used two molecularly distinct IL-1R antagonists, specifically Kineret, a clinically-approved competitive inhibitor of IL-1R, and 101.10, an all-d heptapeptide (sequence: rytvela) non-competitive inhibitor of IL-1R^34,41,42^; this approach was adopted since different pharmacologic agents may exhibit different properties. Our findings reveal an important role for antenatal IL-1β, a major mediator of chorioamnionitis, in causing prolonged ocular inflammation in the offspring resulting in damage to retinal and sub-retinal vasculature, structure and function; these deleterious effects were prevented by antenatal 101.10 to a greater extent than Kineret.

## Methods

### Animals

The use of timed-pregnant CD-1 mice for this study was first approved by the Animal Care Committee of Sainte-Justine’s Hospital following the principles of the Guide for the Care and Use of Experimental Animals developed by the Canadian Council on Animal Care. Animals were ordered from Charles River Inc., all having reached the 11^th^ day of gestation. They were kept in the animal facility for a few days before starting the experiments to let them get used to their new environment. From their arrival to the end of the experiments, they had free access to chow and water, and were kept in a 12:12 light/dark cycle.

### Chemical products

The following products were used: rhIL-1β (#200-01B; PeproTech, Rocky Hill, New Jersey), 101.10 (Elim Biopharmaceuticals, Hayward, California), Kineret (Sobi, Biovitrum Stockholm, Sweden) and RU-486 (Mifepristone; M8046; Sigma-Aldrich, Darmstadt, Germany).

### Animal model of PTB induced by IL-1β

**Figure 1:**
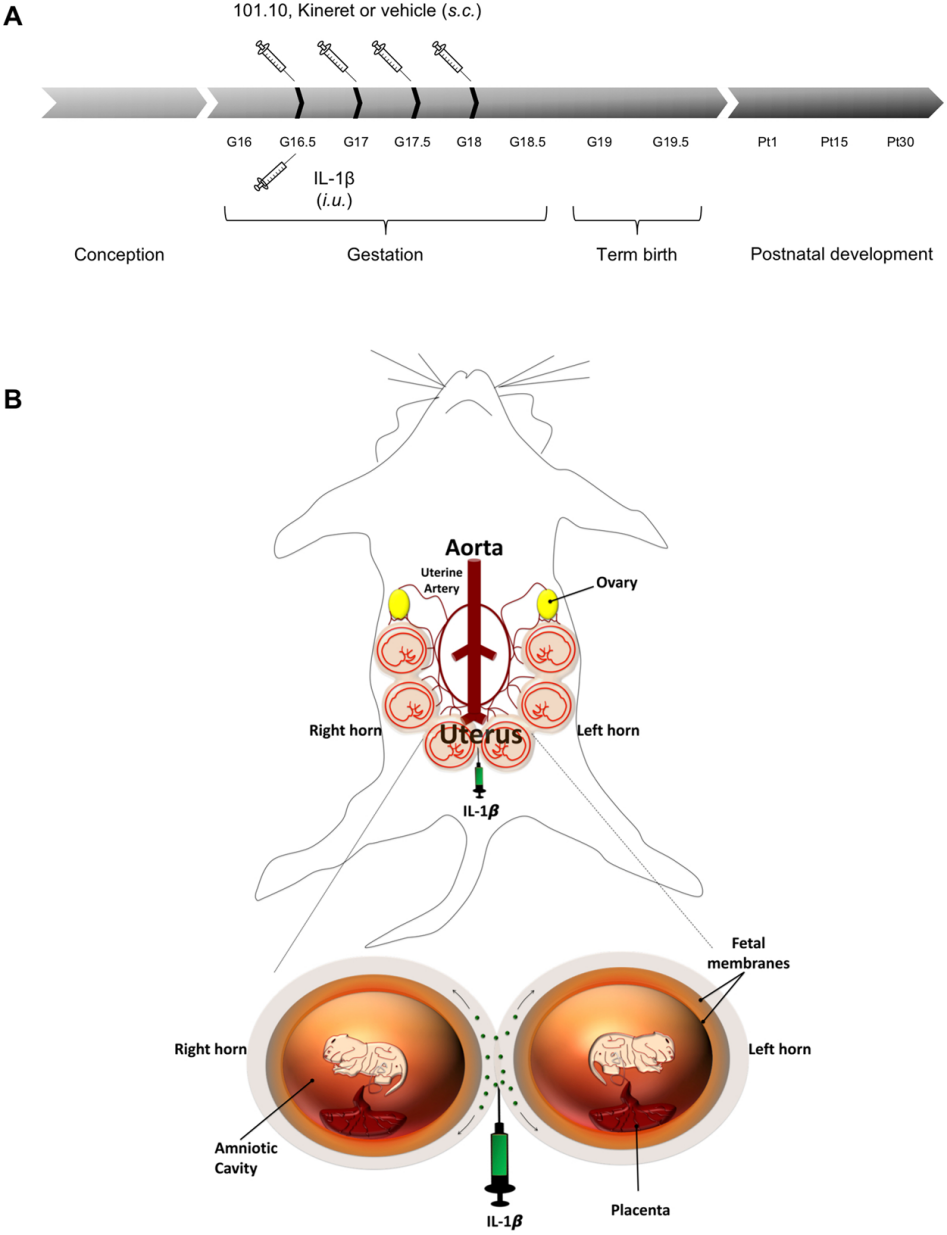
Murine model of inflammation-induced PTB employed for the study. (**A**) IL-1β (1 µg) was administered intrauterine at G16.5, whereas 101.10 (1 mg/Kg/12 h), Kineret (4 mg/Kg/12 h) or vehicle were administered subcutaneously for 2 consecutive days, with the first dose administered 30 min prior to IL-1β. (**B**) Intrauterine injection of IL-1β was made between two foetal membranes, without penetrating the amniotic cavity.

Pregnant CD-1 mice were injected subcutaneously in the neck at gestation day (G) 16.5 with 101.10 (1 mg/Kg/12 h), Kineret (4 mg/Kg/12 h [clinically recommended dose]) or vehicle (Fig. 1A), consistent with previous studies^22^. Half an hour later, mice were anaesthetized with isoflurane with a mask on the muzzle, which was kept during all interventions. With surgical scissors, a 1.5 cm cut was made in the abdomen to expose the uterine horns. An injection of rhIL-1β (1 μg) was made between two foetal membranes as reported^42^, being careful not to penetrate the amniotic cavity using transillumination; a schematic diagram is presented in Fig. 1B. Abdominal muscles were sutured and the skin was stapled shut. Pregnant dams were thoroughly examined every 2 h until term (G19–19.5) to observe the moment of birth and assess the health of newborns.

### Animal model of PTB without inflammation

A single intraperitoneal injection of the progesterone receptor antagonist RU-486 (1 μg/kg) was made at G17. Mice were closely observed until parturition.

### Prenatal tissue collection

To collect the foetal eyes during gestation, 3–5 mice/group were sacrificed at G17, G17.5 and G18. After anaesthesia with isoflurane, they were euthanized with CO_2_ and cervical dislocation. The same method of euthanasia was performed for all mice used in the following experiments. Caesarean sections were performed by widely opening the abdomen with surgical scissors and exposing uterine horns. Foetuses were extracted from their amniotic sacs, decapitated and their eyes were collected and snap-frozen in dry ice. They were preserved at −80 °C for further biochemical analyses.

### Postnatal tissue collection

At birth, 6–8 pups were sacrificed, their eyes collected and quickly processed for biochemical analyses. After term (24 h after G19), 5–7 pups/group were sacrificed at post-term days (to adjust for prematurity) (Pt) 1, Pt 4, Pt 8, Pt 15, Pt 22 and Pt 30 to collect their eyes for histological experiments. The remaining pups were kept alive with their mother until weaning at 3 weeks old. They were then isolated for electrophysiological experiments at Pt 30.

### RNA extraction and Real-Time quantitative Polymerase Chain Reaction (RT-qPCR)

Eyes collected antenatally were submerged in 500 μL of Ribozol (AMRESCO, Solon OH, United States) either after dissection and isolation of retina and sub-retina, or as a whole, to prevent RNA degradation. Samples were then homogenized and RNA was isolated as per manufacturer’s instructions. Using the spectrophotometer Nanodrop 1000, RNA concentration and purity (ratio A260:A280 > 1.6) were measured. For each sample, 500 ng of RNA was converted into complementary DNA (cDNA) using the synthesis kit iScript Reverse Transcription Supermix (Bio-Rad; Hercules CA, United States). Primers were designed with NCBI Primer Blast. Gene expression was quantified with Stratagene MXPro3000 (Stratagene) with SYBR Green Master Mix (Bio-Rad). Gene expression levels were standardized with the universal primer 18 S (Ambion Life Technology; Burlington ON, Canada). Dissociation curves were obtained to verify primer specificity. We analysed the following genes: *Il1b*, *Il6*, *Il8*, *Il12*, *Ccl2*, *Casp1*, *Tnfa*, *Il4*, *Il10* and *Il27*, as previously described^22,28^; primer sequences are detailed in Table 1.

**Table 1:** Forward and reverse sequences of primers used for RT-qPCR gene analysis.

Primers		
Gene	Forward	Reverse
IL-1β	5′-CAACGATGATGCACTTGCAGA-3′	5′-GGAAGGTCCACGGGAAAGAC-3′
IL-6	5′-AGATGAAGGGCTGCTTCCAAA-3′	5′-TCTCTCTGAAGGACTCTGGCT-3′
IL-8	5′-TGCTTTTGGCTTTGCGTTGA-3′	5′-GTCAGAACGTGGCGCTATCT-3′
IL-12	5′-TTCTCACCGTGCACATCCAA-3′	5′-GAGGAGGTAGCGTGATTGACA-3′
CCL2	5′-GCTCAGCCAGATGCAGTTA-3′	5′-TGTCTGGACCCATTCCTTCT-3′
Caspase-1	5′-CACAGCTCTGGAGATCGGTGAA-3′	5′-CCACGGCATGCCTGAATAATG-3′
TNFα	5′-ATGGCCTCCCTCTCATCAGT-3′	5′-TGGTTTGCTACGACGTGGG-3′
IL-4	5′-CCATATCCACGGATGCGACA-3′	5′-CTGTGGTGTTCTTCGTTGCTG-3′
IL-10	5′-AGGCGCTGTCATCGATTTCT-3′	5′-TGGCCTTGTAGACACCTTGGT-3′
IL-27	5′-TGTCCACAGCTTTGCTGAAT-3′	5′-AGGAGGTCCAGGTTCACTCC-3′

### Immuno-enzymology method ELISA

ELISA was performed as documented^22,28^ and as recommended by the manufacturer for the following kits: mouse IL-1β/IL-1F2 Quantikine (#MLB00C; R&D systems), mouse IL-6 Quantikine (#M6000B; R&D systems) and mouse IL-8 (#MBS261967; Mybiosource; recognizes the IL-8 homologue CXCL2). Tissue was equally distributed and briefly lysed in a RIPA solution containing proteases to inhibit protein degradation. Each 50 μL sample was then loaded on a 96-well plate previously embedded with specific primary antibodies, then incubated for 2 hours at room temperature. Then, wells were washed 5 times and incubated for 2 hours with an enzyme-linked polyclonal secondary antibody. After further washes, substrate solution was added. Reaction was stopped after 30 min and plates were read at 450 nm with a wavelength correction of 570 nm.

### Eye collection and fixation

Foetuses and pups (<Pt4) were decapitated or sacrificed with CO_2_ and cervical dislocation (>Pt7). By pressing around the orbits with small scissors, eyes were pulled out of the skull and collected by pinching the optic nerve. The cornea was pierced with a syringe and the eyes were fixated for 20–30 min in PFA 4%, then transferred in PBS and preserved at 4 °C until they were processed for flat mounts or cryosections.

### Immunofluorescence

Eyes were dissected to withdraw the cornea and the lens; the rest was fixated in sucrose 30% for 24 hours at 4 °C. They were then imbedded in Frozen Section Compound (FSC 22 Clear), snap-frozen in dry ice and sectioned with a cryostat at a thickness of 14 μm. Classical immunofluorescence technique was used with antibodies against lectin (Bandeiraea simplicifolia, Sigma-aldrich; 1:100), Iba-1 (ab5076, Abcam; 1:500) and Dapi (Invitrogen; 1:5000). Fluorescent secondary antibody was applied for the Iba-1 labelling (Alexa fluor 488, Abcam; 1:200). Images were taken with a confocal microscope (TCS SP2, Leica Microsystems) using a 20X objective.

### Retinal and choroidal flat mounts

For each group, eyes of 5 mice at Pt1, Pt4, Pt8, Pt15, Pt21 and Pt30 were dissected as reported^43^, to isolate the retina and choroid/retinal pigment epithelium (RPE) complex. Flat mounts were then labelled with a fluorescent antibody against lectin (Sigma-aldrich; 1:100) and Iba-1 (ab5076, Abcam; 1:500). Fluorescent secondary antibody was applied for the Iba-1 labelling (Alexa fluor 488, Abcam; 1:200). On a microscope slide, 4 cuts were performed with a scalpel to flatten the retina and choroid/RPE complex. With a paintbrush soaked with PBS, hyaloid vessels were removed. Images were obtained in confocal microscopy using a 20 × (lectin) or 100 × (Iba-1) objective. For Iba-1 imaging, the confocal was focused on the superficial retinal vascular layer at Pt 1 (corresponding to the nerve fibre layer [NFL]) and below the intermediate retinal vascular layer at Pt 15 and Pt 30 (corresponding to the INL)^44^.

### Histological quantification

Vascular surface was measured with ImageJ and expressed in percentage of total retinal surface, and vessel density was analysed using ImageJ software in the mid-periphery of the retina, as described^43^. Briefly, lectin immunolabelling (blood vessels, showed as red on the retina) was isolated from other structures using the colour deconvolution tool in ImageJ. The detection threshold was established to reduce artefacts and a semi-quantitative comparison of vascular density was performed. Iba-1 positive (Iba1^+^) cells were counted using the ImageJ software in the central retina at Pt1 and in the mid-periphery at Pt15 and 30, as reported^45^.

In order to measure the thickness of the different retinal layers and of the choroid, ImageJ software was used to draw a line across the region to measure. Measures were taken at the central, middle and periphery of the retina and were repeated to obtain results that were more representative. A ratio was calculated for each eye to make a comparison between the groups. The retinal thickness was measured between the outer nuclear layer (ONL) and the ganglion cell layer (GCL) using the same method than the choroid, as we reported^21,39,46^.

### Electroretinography

Electroretinogram (ERG) recording was performed with Espion ERG diagnosys machine with ColorDome Ganzfeld stimulator (Diagnosys LLC, Lowell, MA), as reported^39^. Mice were dark-adapted overnight and anesthetized prior to ERG with an intraperitoneal injection of Ketamine (100 mg/kg) and Xylazine (20 m/kg) mixture and mouse body temperature was kept at 37 °C using heated pad. DTL Plus electrodes (Diagnosys LLC) were positioned at the surface of the cornea after pupil’s dilatation with drops of 1% atropine and 2.5% phenylephrine (Alcon) and flash ERG were measured. Scotopic responses were simultaneously recorded at light intensity 0.9 cd-s/m². ERG was performed under red light in a dark room. After recording, eye hydration was carefully verified, and mice were kept at 37 °C until they awoke.

### Statistical analysis

The term dam/group refers to the dams per group regardless of number of progeny (pups) of at least 1; hence n = 1 more for a single dam (with ≥1 progeny). Parametric analysis was performed since the power analysis is generally greater for continuous parametric variables, and given the spread of data between groups^47^. Comparisons between two variants was analysed by t-test. Comparisons between several groups were performed using one-way variance analysis (ANOVA); Dunnett’s multiple comparison method was used when many treatments were compared with one control. The value p < 0.05 was considered statistically significant. Data is presented as means ± S.E.M^47^.

### Data availability

Data sets generated during this study can be obtained from the corresponding author upon request.

## Results

### *In utero* exposure to IL-1β causes an acute perinatal inflammatory response in the foetal retina and sub-retina

To study the retinal and sub-retinal vascular development in foetuses and pups after exposure to IL-1β *in utero*, we employed an established murine model of IL-1β-induced chorioamnionitis^34,42^. As expected, intrauterine IL-1β (G16.5 days) shortened gestation and induced marked neonatal mortality; 101.10 prolonged gestation to term and markedly augmented foetal survival (Suppl. Fig. 1); whereas Kineret exerted no protective effect, as previously reported^34^.

**Figure 2:**
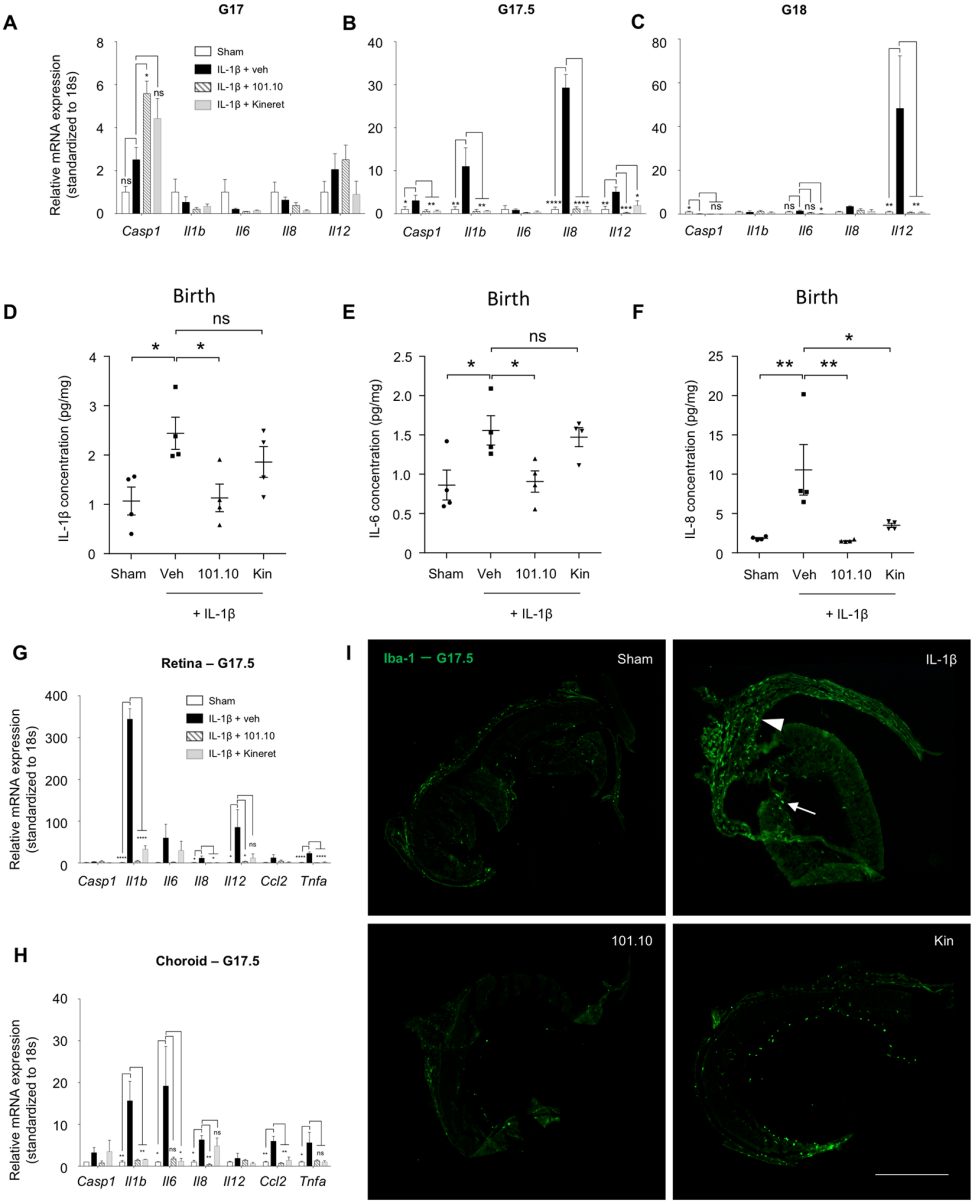
Inflammatory response in the retina and sub-retina of the foetus and newborn. (**A**–**C**) Foetal eyes were collected at G17 (**A**), G17.5 (**B**) and G18 (**C**) after *in utero* exposure to IL-1β to perform quantitative PCR. Results are relative to 18 S and plotted as fold change vs. the control groups. n = 3–8 dams/group; 4 foetal eyes per sample. **(D**–**F)** Cytokine levels in eyes of newborns exposed to the indicated treatments *in utero* (Fig. 1). n = 4 dams/group; 4 eyes per sample. (**G**–**H**) Quantitative PCR of IL-1, IL-6 and IL-8 performed on isolated retina (**G**) and choroid (**H**) collected on foetuses at G17.5; n = 3 dams/group; 10 retinas or sub-retinas per sample. (**I**) Iba-1-stained cryosections of retina from foetuses at G17.5 exposed to the indicated treatments *in utero*; n = 3 foetuses/group. Kin refers to Kineret. Scale bar, 300 μm. Values are presented as mean ± S.E.M. *p < 0.05, **p < 0.01, ***p < 0.001 by one-way ANOVA with Dunnett’s post-analysis.

Eyes (undissected) of foetuses exhibited increased mRNA expression of pro-inflammatory mediators with a variable profile depending on timing after exposure to IL-1β-induced chorioamnionitis (Fig. 2A–C); at birth, increased protein levels of IL-1β, IL-6 and IL-8 were detected in the eyes (Fig. 2D–F); these were normalized by 101.10, but not by Kineret with the exception of IL-8 attenuation. Interestingly, early (at G17.5) IL-1β-induced expression of anti-inflammatory *Il27* was suppressed by Kineret but preserved by 101.10; while expression of *Il10* was diminished by both Kineret and 101.10 (Suppl. Fig. 2). To better localize the intraocular inflammatory response, we isolated the retina and choroid of foetuses. IL-1β triggered early (G17.5) mRNA expression of *Il1b*, *Il6*, *Il8*, *Il12*, *Ccl2* and *Tnfa* in retina more than in choroid (Fig. 2G,H), which was associated with increased intra-ocular accumulation of Iba1^+^ cells (activated macrophage/microglia)^48^ (Fig. 2I), as reported in inflammation-associated retinopathy^49^; this Iba1^+^ cell accumulation is clearly distinct from the relatively low level of Iba1^+^ cells in control animals wherein it plays a role in normal retinal development^50^. Notably, antenatal 101.10 and Kineret significantly attenuated marked IL-1β-elicited cytokine induction and Iba1^+^ cell accumulation (Fig. 2G–I).

### Iba1^+^ cell accumulation persists post-natally in retina and choroid of newborns subjected *in utero* to inflammation

**Figure 3:**
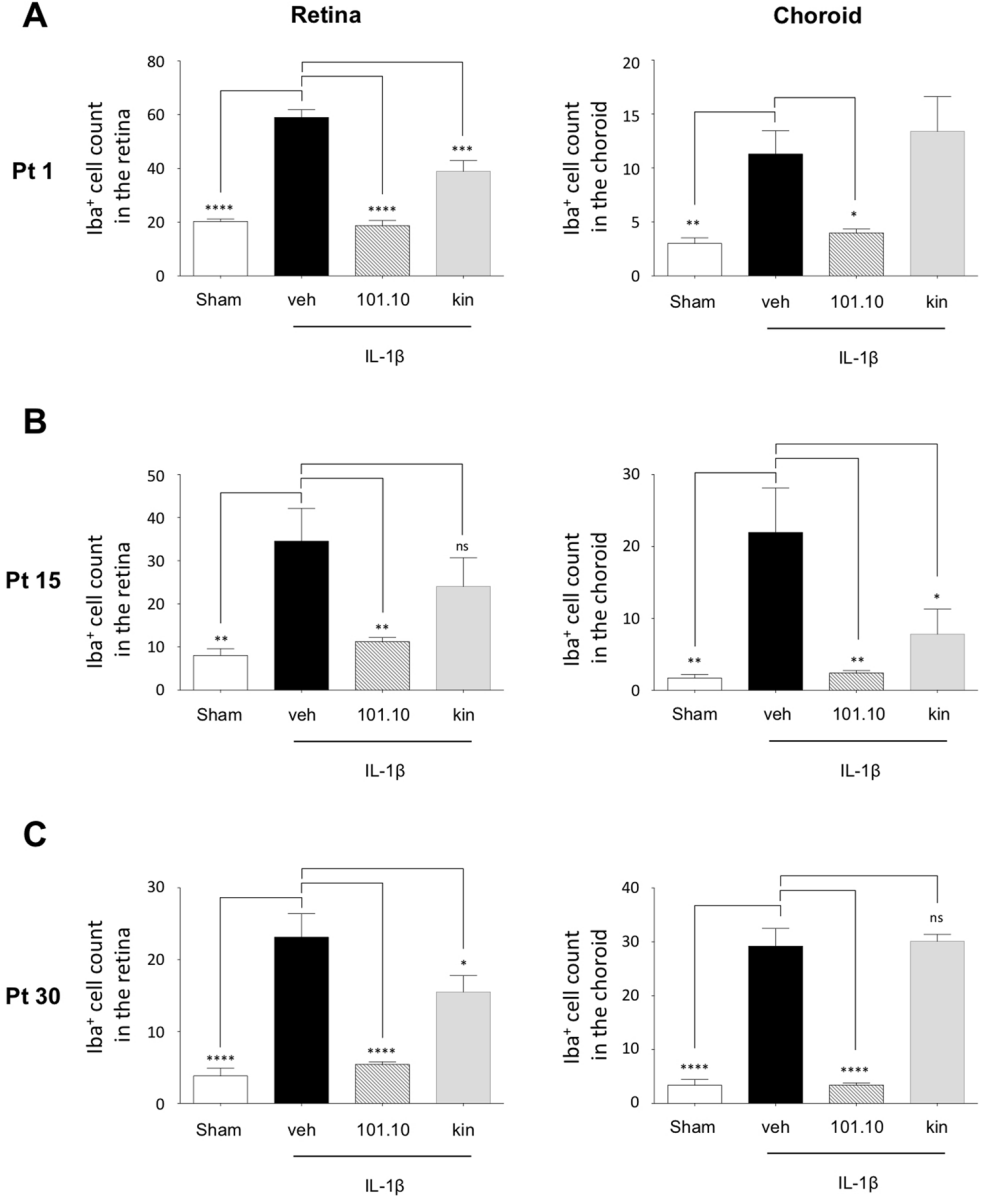
Infiltration of immune cells in eyes during development of the pups. (**A**–**C**) Quantification of iba-1^+^ cells observed on retinal and choroidal flat mounts at Pt 1 (**A**), Pt 15 (**B**) and Pt 30 (**C**). n = 3–6 pup/group for each time point. Values are presented as mean ± S.E.M. *p < 0.05, **p < 0.01, ***p < 0.001, ****p < 0.0001 by one-way ANOVA with Dunnett’s post-analysis.

Abnormally high numbers of Iba1^+^ cells in retina over extended duration can affect vascular development and in turn cause long-term deficits of retinal function^37^. Since perinatal inflammation resulting in retinopathy is associated with prolonged inflammation^37^ with IL-1β-dependent microglial accumulation^38^, we determined if Iba1^+^ cells persisted in retina and choroid of animals exposed to IL-1β-induced chorioamnionitis. We detected a 3-fold increase in Iba1^+^ cell density on post-term (Pt) day 1 in eyes of animals exposed to antenatal IL-1β, which persisted on postnatal days 15 and 30 in retina and choroid (Fig. 3 and Suppl Fig. 3), consistent with previous observations related to early postnatal inflammation^37^; coincidentally, *Il1b* expression was increased from Pt 1 to Pt 15, and subsided by Pt 30 (Suppl. Fig. 4A) as inflammation resolved (in presence of Iba1^+^ cells)^51,52^. Inflammatory cell accumulation and *Il1b* expression was normalized by antenatal 101.10 (Fig. 3 and Suppl Figs 3 and 4A).

### Chorioamnionitis leads to persistent ocular inflammation in newborn offspring associated with impaired retinal vascular development and choroidal involution

**Figure 4:**
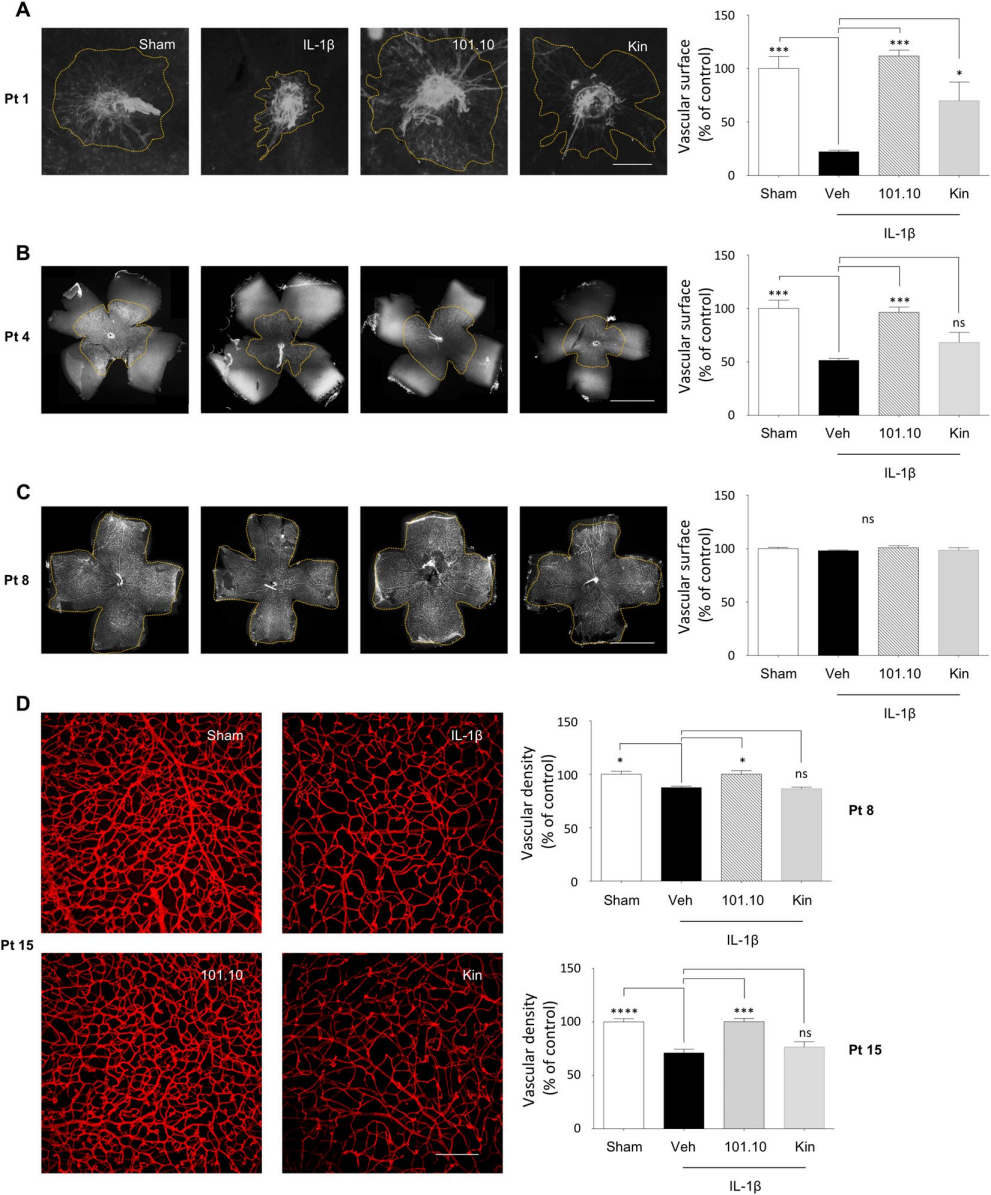
Delay of retinal vessel growth in progeny. (**A–C**) Lectin-stained flat-mounts of retinas from pups at Pt 1 (**A**), Pt 4 (**B**) and Pt 8 (**C**) previously exposed *in utero* to the indicated treatments (Fig. 1). Images are representative of 3 to 5 separate pups per treatment group. Dotted lines depict the vascular front. Scale bar for A, 1500 μm; scale bar for B, 2500 μm; scale bar for C, 3000 μm. Right panels show quantification of the vascular area, n = 3–5 dams/group. (**D**) Magnification of lectin-stained flat-mounts of retinas from pups at Pt15 showing vascular density. Images are representative of 5 to 8 separate pups per treatment group. Scale bar, 150 μm. Right panel shows quantification of the vascular area at Pt8 and Pt15, n = 5–8 dams/group. Values are presented as mean ± S.E.M. *p < 0.05, ***p < 0.001 by one-way ANOVA with Dunnett’s post-analysis.

Based on evidence of increased inflammation in retina and choroid in early weeks after birth we evaluated the impact of chorioamnionitis on retinal vessel growth and choroidal thickness in pups. Retinal vascularized surface area during the first postnatal week was reduced in animals subjected to IL-1β-induced chorioamnionitis (consistent with previous reports^53^) (Fig. 4A,B), but by Pt 8 retinal surface was fully vascularized in all groups as vessels reached the periphery (Fig. 4C); concordantly, *Il1b* expression was only marginally increased at Pt 8 (p < 0.06 vs sham-treated; Suppl. Fig. 4A). On the other hand, a small decrease in pan-retinal vascular density was detected in mice exposed to chorioamnionitis at Pt 8, which corresponds to the period wherein intra-retinal vessels are actively forming. This hypo-vascularization was aggravated at Pt 15 (Fig. 4D) when *Il1b* (mRNA) expression markedly rose (Suppl. Fig. 4A); ultimately retinal vascularization normalized at Pt 30 (Suppl. Fig. 4B) along with *Il1b* (mRNA) expression (Suppl. Fig. 4A). Antenatal 101.10 fully rescued retinal vascularization at all ages analysed, whereas Kineret was only partially effective at Pt 1 and ineffective subsequently at Pt 4, Pt 8 and Pt 15 (Fig. 4).

**Figure 5:**
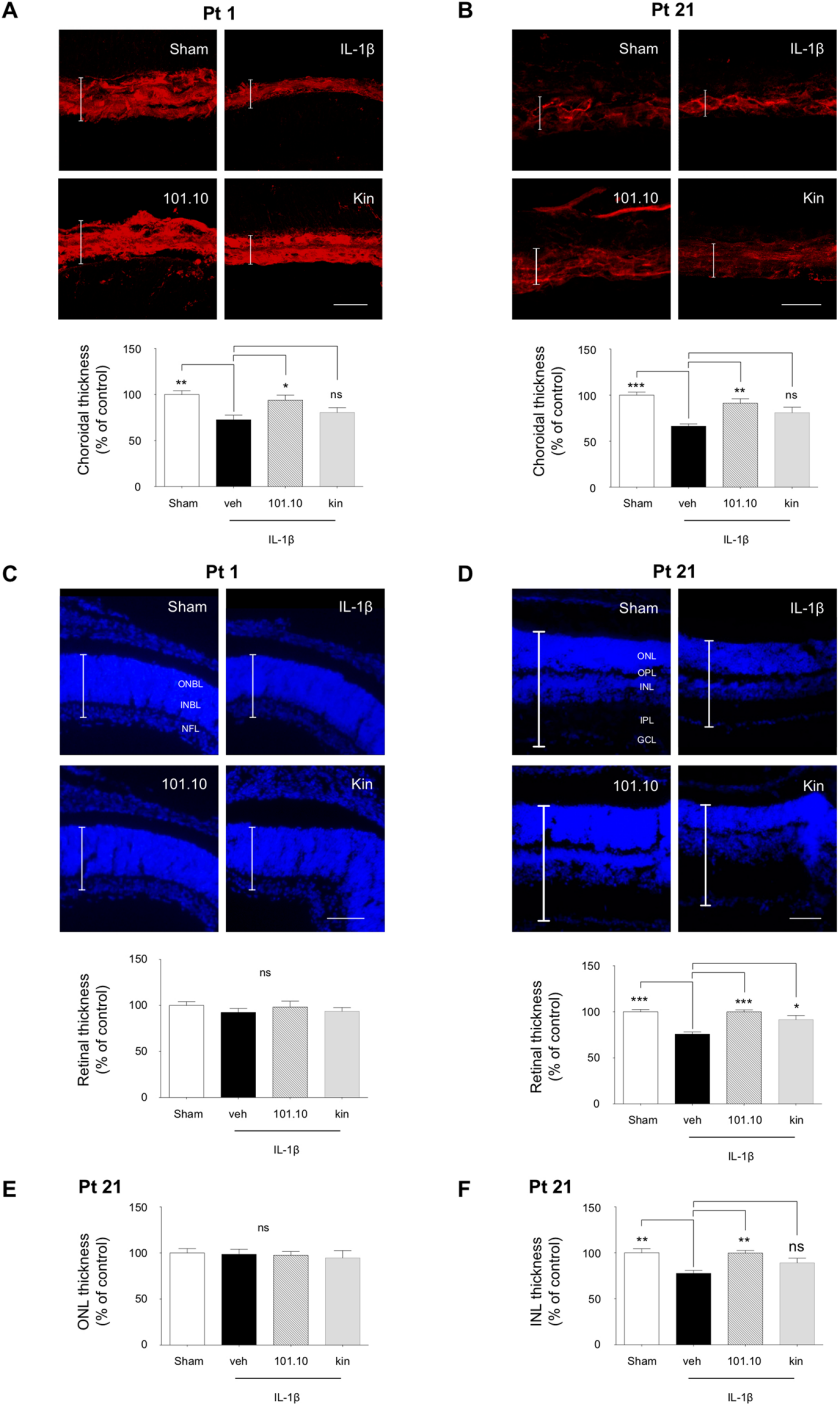
Choroidal thinning precedes retinal degeneration and thinning of the inner nuclear layer. (**A–B)** Representative images (top panels) and quantification (bottom panels) of lectin-stained cross-sections of choroids from pups at Pt 1 (**A**) and Pt 21 (**B**) previously exposed to the indicated treatments *in utero* (Fig. 1). Vertical bars represent the average choroidal thickness. Scale bar for A, 30 μm; scale bar for B, 50 μm. n = 3–4 pups/group. (**C**,**D**) Representative images (top panels) and quantification (bottom panels) of DAPI-stained cross-sections of retinas from pups at Pt1 (C) and Pt21 (**D**) previously exposed to the indicated treatments *in utero* (Fig. 1); n = 3–4 pups/group. Vertical bars represent the average retinal thickness. Scale bar for C, 250 μm; scale bar for B, 500 μm. (**E**,**F)** Quantification of DAPI-stained cross-sections of the ONL (**E**) and INL (**F**) from the same retinas measured in D. Values are presented as mean ± S.E.M. *p < 0.05, **p < 0.01, ***p < 0.001 by one-way ANOVA with Dunnett’s post-analysis. ONBL, outer neuroblastic layer; INBL, inner neuroblastic layer; NFL, nerve fibre layer; ONL, outer nuclear layer; OPL, outer plexiform layer; INL, inner nuclear layer; IPL, inner plexiform layer; GCL, ganglion cell layer.

Since the choroid is an exclusive blood supply to the outer retina and its involution was recently linked to ROP and retinal functional deficits^39,46^, we measured choroid thickness. As anticipated, choroid thickness was normal soon after IL-1β treatment (at G17.5) (Suppl. Fig. 4C). By Pt 1 choroidal thinning was observed; these changes persisted beyond the neonatal period (at Pt 21) (Fig. 5A,B). Antenatal 101.10, but not Kineret, preserved choroidal (normal) thickness.

To demonstrate that the effects of IL-1β are unrelated to prematurity *per se*, we showed that PTB induced with the progesterone receptor antagonist RU486 did not significantly affect ocular cytokine profile or postnatal vascular development (Suppl. Fig. 5), inferring an important role for perinatal inflammation on the latter.

### Retinal structural and functional deficits in offspring secondary to gestational tissue-triggered inflammation

**Figure 6:**
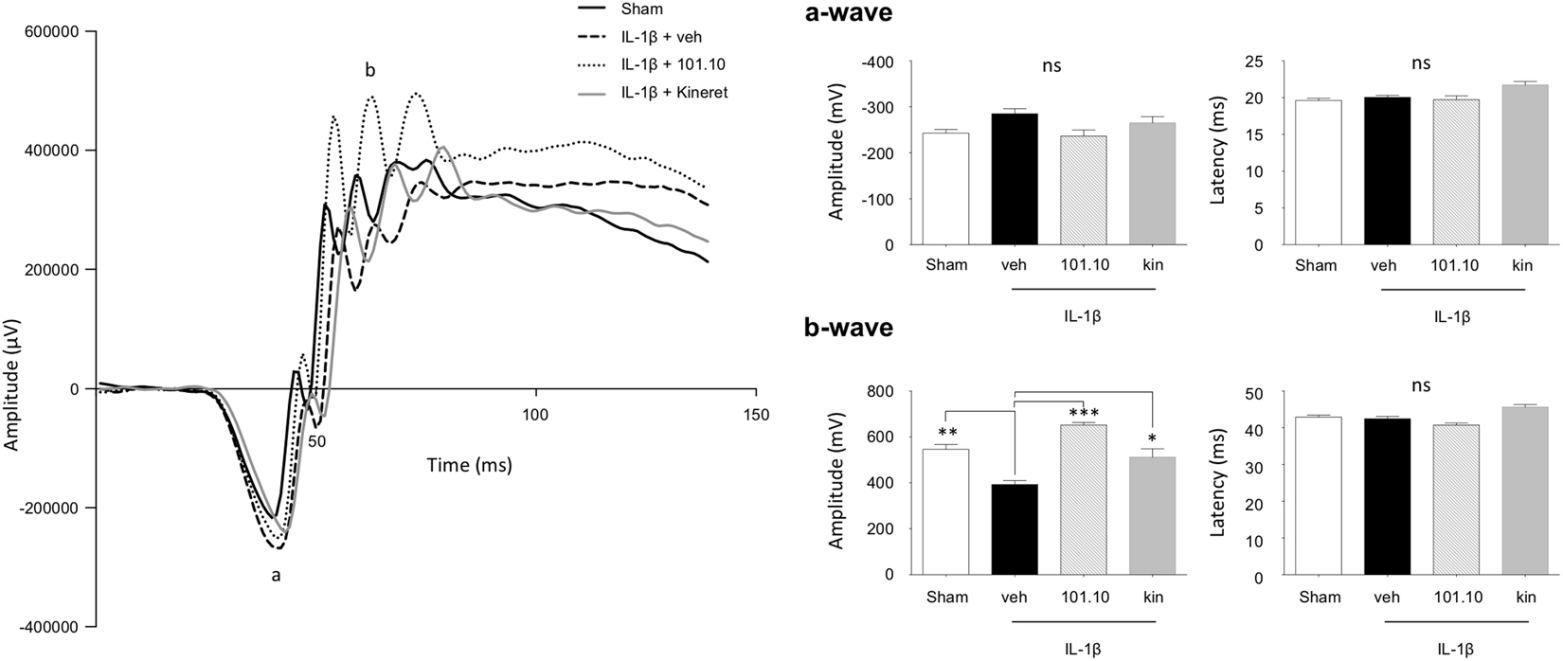
Electroretinogram readings after completion of ocular development. (**A**) Representative ERG response of pups at Pt 30 previously exposed to the indicated treatments *in utero* (Fig. 1). (**B,C**) a-wave amplitude (**B**) and latency (**C**) of scotopic ERGs of corresponding animals. (**D**,**E**) b-wave amplitude (**D**) and latency (**E**) of scotopic ERGs of corresponding animals. n = 8–22 pups/group. Values are presented as mean ± S.E.M. *p < 0.05, **p < 0.01, ***p < 0.001 by one-way ANOVA with Dunnett’s post-analysis.

Retinal development depends upon normal blood supply^54^, such that both retinal structure and function are affected by vasculopathy as seen in ROP^21,39^. Accordingly, we measured retinal thickness from the outer neuroblastic layer to the nerve fibre layer at G17.5 and Pt 1, and between the ONL and the GCL at Pt 21 (of the same eyes used to assess choroidal thickness). As expected^39^, full retinal thickness was not affected within the first 2 weeks after antenatal-induced inflammation (Fig. 5C). By Pt 15, following retinal vasculopathy (Fig. 4), there was a tendency for decreased thickness of the retina (Suppl. Fig. 6A), which was significantly manifested at Pt 21 (Fig. 5D), and was largely attributed to thinning of the inner nuclear layer (Fig. 5F and Suppl. Fig. 6B); this resulted in a corresponding decreased b-wave amplitude (largely contributed by the inner nuclear layer) (Fig. 6). The ONL thickness and corresponding a-wave amplitude remained intact at the end of the first postnatal month (Figs 5E and 6), as reported in ROP-associated choroidopathy^39^. Retinal morphometry and inner retinal function were fully preserved by antenatal 101.10, but only partially by Kineret.

## Discussion

Gestational inflammation is an independent risk factor for the development of neonatal morbidities, including to the eye^11,16,19^. Yet, the identification of a causal factor playing a pivotal role remains a challenge. Studies have suggested that the pro-inflammatory cytokine IL-1β is involved in the pathophysiology of many perinatal complications^55,56^. A link between chorioamnionitis and neonatal brain injury is described^19^, and associated with vascular damage^42^. Of relevance, IL-1β plays a critical role in central nervous system insult during the perinatal period^42,57,58^. In retinal injury the role of IL-1β has mostly been ascribed to inflammation resulting from exposure to hyperoxia^37–40^, an important predisposing factor to ROP. However, there is still no evidence demonstrating a direct role for IL-1β in retinal/sub-retinal injury of the immature subject. Given that retinal damage represents a major complication of prematurity, itself often associated with inflammation, we set out to discriminate the effects of antenatal (intra-uterine) IL-1β-triggered inflammation on retinal and sub-retinal vascular development in offspring during the perinatal period and adolescence; in this process we also determined the efficacy of 101.10 (compared to Kineret). By inducing utero-placental inflammation as reported^34,42^ with intra-uterine IL-1β (which does not cross the placental barrier^59^), we found a prolonged inflammation in the eye that begins ante-natally and ensues in choroidal and retinal vascular, structural and ultimately sustained functional deficits; these detrimental changes were prevented by antenatal 101.10 (but not [recommended dose of] Kineret).

The cascade of inflammatory mediators amplified by IL-1β exerts cytotoxicity on its own. As alluded to above macrophage/microglial invasion is a major contributor of IL-1β generation which in turn exerts endothelial cytotoxicity via neuronally-generated Semaphorin3A^38^, and causes direct neurotoxicity including to photoreceptors^60,61^. In cultured retinal endothelial cells, chronic exposure (5 days) to IL-1β leads to increased caspase-3 activity and apoptosis^36^. Concordantly, inhibition of IL-1β signalling using pharmacological or genetic approaches decreases caspase activity and apoptosis in retina, and prevents microvascular degeneration in hyperglycaemia-induced retinopathy models^62^. Comparably, IL-12 is a cytokine produced by macrophages, neutrophils, and other inflammatory cells, and acts by triggering T-cell differentiation^63^; IL-12 exhibits antiangiogenic properties^64^, and has been associated with serious ocular diseases including uveitis^65^. Interestingly, IL-1-induced IL-12 retinal production was inhibited by 101.10 which may represent an additional mechanism through which 101.10 protects retinal vasculature. The role of TNF in cytotoxicity is more complex as it depends on the receptor it acts upon which may convey opposing actions^66,67^.

In addition to retinopathy, choroidopathy has lately also been regularly observed in subjects formerly afflicted with ROP as recently reviewed^68,69^; and this feature was recently reproduced in models of ROP^39^. Involution of the choroid was found to be sustained and subsequently led to retinal pigment epithelium and photoreceptor degeneration^39^. In this context, IL-1β exerts a major contribution to choroidal thinning of the newborn subjected to perinatal oxidative stress, which in turn results in long-term injury to the sub- and outer retina. In line with this concept, we observed sustained presence of inflammatory cells post-natally, resulting in choroidal thinning as early as Pt 1, which we had found to predispose to subsequent photoreceptor injury. Together, current and previous observations^39^ related respectively to antenatal and postnatal inflammation and ensued retinal damage, reveal efficacy of anti-IL-1β treatment. Our findings are consistent with brain neuronal cell death of pups exposed to IL-1β-induced chorioamnionitis^42^. Hence, chorioamnionitis triggers an inflammatory cascade that spreads to the foetus causing retinal and choroidal injury.

An important feature reported herein is the long-term deficit in retinal structure (inner nuclear layer thinning) and function caused by exposure to IL-1β *in* *utero*, despite recovery of retinal vascular density at Pt 30 which coincided with abated IL-1β expression, consistent with diminished expression of inflammatory cytokines in residing macrophages upon resolution of the pro-inflammatory state^51,52^. Inner retinal functional deficits (depicted by b-wave amplitude suppression) are well described in former ROP subjects and believed to result prominently from retino-vascular injury^21,68^. Although we also observed early involution of the choroid upon exposure to utero-placental inflammation, time is needed until developmentally increased photoreceptor metabolism can no longer be supported by insufficient O_2_ and nutrient supply from the damaged choroid, resulting in photoreceptor damage^39^. Hence, we surmise a gradual degeneration of photoreceptors (and retinal pigment epithelium) after exposure to antenatal inflammation. Together, the retinal and sub-retinal damage observed in response to antenatal IL-1β are indicative of an important role for inflammation, found to be prolonged.

A relevant aspect of this study applies to the efficacy of 101.10 compared to Kineret in protecting the choroid and retina. We recently showed that inhibition of IL-1 receptor using a novel small peptide labelled 101.10 was fully effective in preventing PTB, improving survival and preserving foetal lung, gut and brain integrity^42^; whereas current commercially available IL-1 receptor antagonist (namely Kineret) were mostly ineffective. These findings represented an unparalleled therapeutic which improved foetal/newborn outcome. Kineret is a competitive antagonist of the IL-1 receptor. Doses utilized corresponded to those clinically recommended (4 mg/kg/dose). Although higher doses of Kineret are more effective, a 7-fold-increase in dosage is needed to augment gestation and ensuing foetal maturation^42^, which would seriously compromise immune response, an established side effect of Kineret. As a competitive antagonist of IL-1 receptor Kineret suppresses both JNK/p38/c-jun/AP-1 and NF-κB pathways; whereas 101.10 acts as a modulator that biases IL-1R-induced signal transduction resulting in inhibition of the JNK/p38/c-jun/AP-1 pathway, while desirably preserving the NF-κB pathway^23,34^, important for immune vigilance^70,71^. Along these lines, in contrast to Kineret, 101.10 maintained activity of some endogenous anti-inflammatory cytokines, notably IL-27 known to neutralize macrophage activity^72^, thus augmenting its anti-inflammatory activity; interestingly, IL-10 release is p38-dependent^73^ and IL-27 generation is NF-kB-dependent^74^, consistent with signalling actions of 101.10 and Kineret. Another disadvantage of Kineret applies to its relatively large size (17.5 kDa) curtailing passage across the placental barrier to sufficiently block inflammation in the foetal compartment^59^; whereas 101.10 is a small molecule (0.85 kDa) that can distribute in gestational tissues^42^, consistent with its preferred antenatal efficacy in suppressing detrimental inflammation and preserving foetal organ integrity.

In summary, this study unveils an unprecedented key role for IL-1β during gestation in the development of long-lasting inflammation and injury to the eye of the progeny, independently of prematurity *per se*. Antenatal inflammation as seen in chorioamnionitis induces damage to the choroid and retinal vasculature, structure and function, all of which is remarkably preserved by the small IL-1 receptor modulator 101.10. 101.10 could represent a novel therapeutic approach not only to tackle PTB^34^ but also in preventing major foetal/neonatal organ injury including to the eye of premature subjects exposed to antenatal inflammation.

## Electronic supplementary material

Supplementary data
