# Author Correction: Antenatal IL-1-dependent inflammation persists postnatally and causes retinal and sub-retinal vasculopathy in progeny

**DOI:** 10.1038/s41598-020-63705-1

**Published:** 2020-04-15

**Authors:** Alexandra Beaudry-Richard, Mathieu Nadeau-Vallée, Élizabeth Prairie, Noémie Maurice, Émilie Heckel, Mohammad Nezhady, Sheetal Pundir, Ankush Madaan, Amarilys Boudreault, Xin Hou, Christiane Quiniou, Estefania Marin Sierra, Alexandre Beaulac, Gregory Lodygensky, Sarah A. Robertson, Jeffrey Keelan, Kristina M. Adams Waldorf, David M. Olson, Jose-Carlos Rivera, William D. Lubell, Jean-Sebastien Joyal, Jean-François Bouchard, Sylvain Chemtob

**Affiliations:** 1 Departments of Pediatrics, Ophthalmology and Pharmacology, CHU Sainte-Justine Research Centre, Montréal, Canada; 2 Department of Pharmacology, Université de Montréal, Montréal, Canada; 3 Department of Pharmacology and Therapeutics, McGill University, Montréal, Canada; 4 Department of Obstetrics and Gynaecology, University of Adelaide, Adelaide, South Australia 5005 Australia; 5 Div Obstetrics & Gynaecology, University of Western Australia King Edward Memorial Hospital, Perth, Australia; 6 Department of Obstetrics & Gynaecology, University of Washington, Seattle, WA USA; 7 Departments of Obstetrics and Gynaecology, Pediatrics and Physiology, University of Alberta, Edmonton, AB Canada; 8 Department of Ophthalmology, Maisonneuve-Rosemont Hospital Research Centre, Montréal, Canada; 9 Department of Chemistry, Université de Montréal, Montréal, Canada; 10 School of Optometry, Université de Montréal, Montréal, Canada

Correction to: *Scientific Reports* 10.1038/s41598-018-30087-4, published online 08 August 2018

The original version of this Article contained a typographical error in the spelling of the author Kristina M. Adams Waldorf, which was incorrectly given as Kristina Adams-Waldorf. This has now been corrected in the PDF and HTML versions of the Article, and in the accompanying Supplementary Data file.

