# Supplementary material for: Antenatal IL-1-dependent inflammation persists postnatally and causes retinal and sub-retinal vasculopathy in progeny
Source: Sci Rep. 2018 Aug 8;8:11875. doi: 10.1038/s41598-018-30087-4 (PMC6082873; doi:10.1038/s41598-018-30087-4)
Supplement: Supplementary file 1 — Supplementary data [file 41598_2018_30087_MOESM1_ESM.pdf]

Title: **Antenatal IL-1-dependent inflammation persists postnatally and causes retinal and sub-retinal vasculopathy in progeny**

Authors and affiliations.
Alexandra Beaudry-Richard[1], Mathieu Nadeau-Vallée[1,2], Élizabeth Prairie[1], Noémie Maurice[1], Émilie Heckel[1], Mohammad Nezhady[1], Sheetal Pundir[1], Ankush Madaan[1,3], Amarilys Boudreault[1], Xin Hou[1], Christiane Quiniou[1], Estefania Marin Sierra[1,3], Alexandre Beaulac[1], Gregory Lodygensky[1], Sarah A. Robertson[4], Jeffrey Keelan[5], Kristina M. Adams Waldorf[6], David M. Olson[7], Jose-Carlos Rivera[8], William Lubell[9], Jean-Sebastien Joyal[1,2,3], Jean-François Bouchard[10] and Sylvain Chemtob[1,2,3]
[1]Departments of Pediatrics, Ophthalmology and Pharmacology, CHU Sainte-Justine Research Centre, Montréal, Canada; [2]Department of Pharmacology, Université de Montréal, Montréal, Canada; [3]Department of Pharmacology and Therapeutics, McGill University, Montréal, Canada; [4]Dept of Obstetrics and Gynaecology, University of Adelaide, Adelaide, South Australia, 5005, Australia; [5]Div Obstetrics & Gynaecology, University of Western Australia King Edward Memorial Hospital, Perth, Australia; [6]Dept of Obstetrics & Gynaecology, University of Washington, Seattle, WA, USA; [7]Departments of Obstetrics and Gynaecology, Pediatrics and Physiology, University of Alberta, Edmonton, AB, Canada; Department of Ophthalmology, [8]Research Centre of Maisonneuve-Rosemont Hospital; [9]Department of Chemistry, Université de Montréal, Montréal, Canada; [10]School of Optometry, Université de Montréal, Montréal, Canada.

Supplementary figure 1

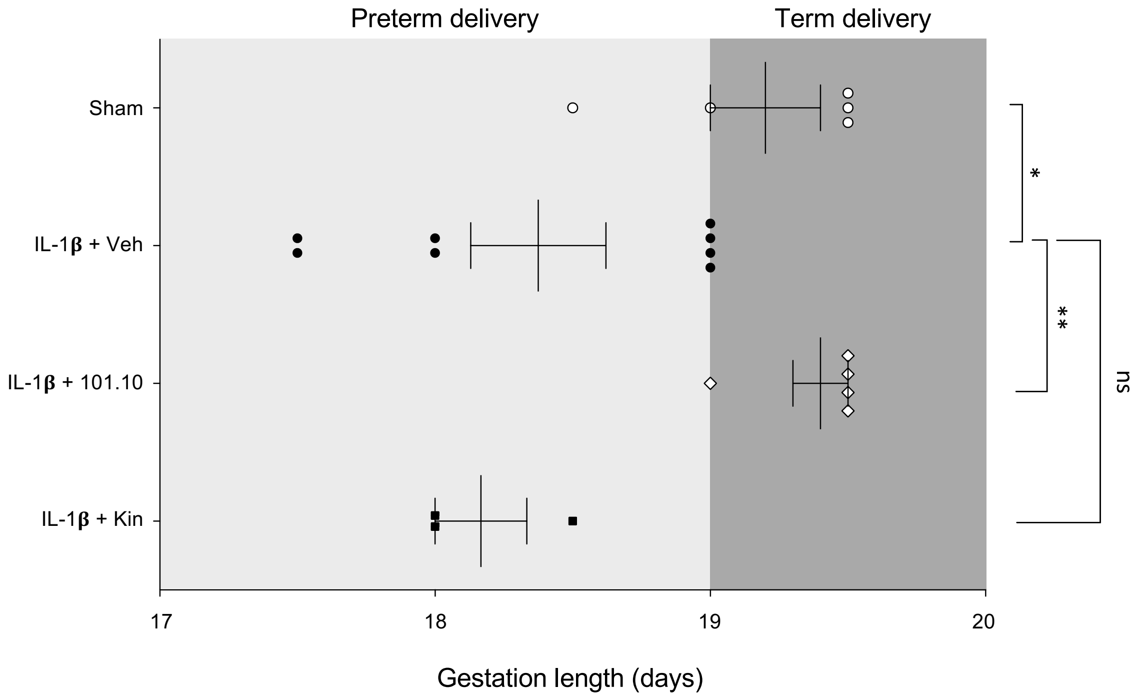

Supplemental figure 1. IL-1β-induced PTB gestation length. Gestation length of dams treated with the indicated treatments (Fig. 1); n=5-8 dams/group. Values are presented as mean ± S.E.M. *p<0.05, **p<0.01, ***p<0.001 by one-way ANOVA with Dunnett's post-analysis.

Supplementary figure 2

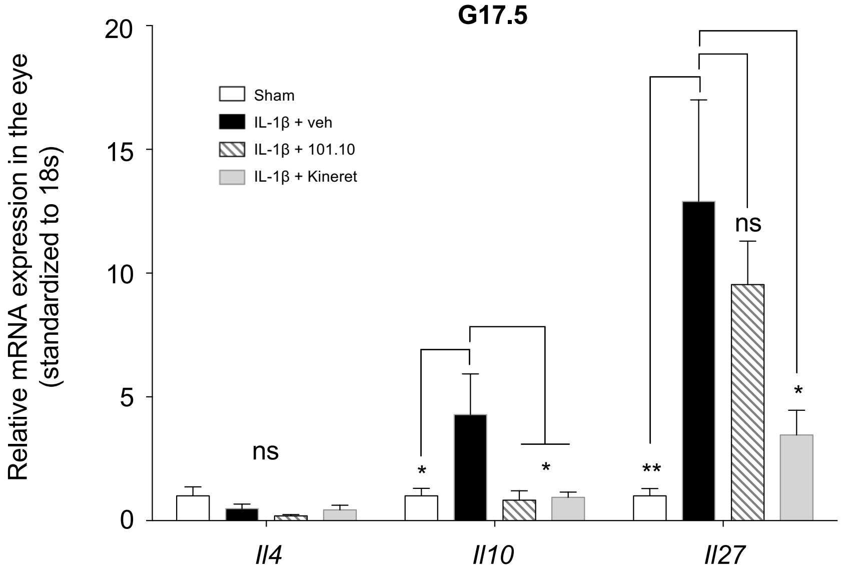

Supplemental Figure 2. Anti-inflammatory response in foetal eyes. Foetal eyes were collected at G17 after *in utero* exposure to the indicated treatments (Fig. 1) to measure *Il4, Il10* and *Il27* mRNA (by PCR). Results are relative to 18S and plotted as fold change vs. the control groups. n=4-5 dams/group; 4 foetal eyes per sample. Values are presented as mean ± S.E.M. *p<0.05, **p<0.01 by one-way ANOVA with Dunnett's post-analysis.

Supplementary figure 3

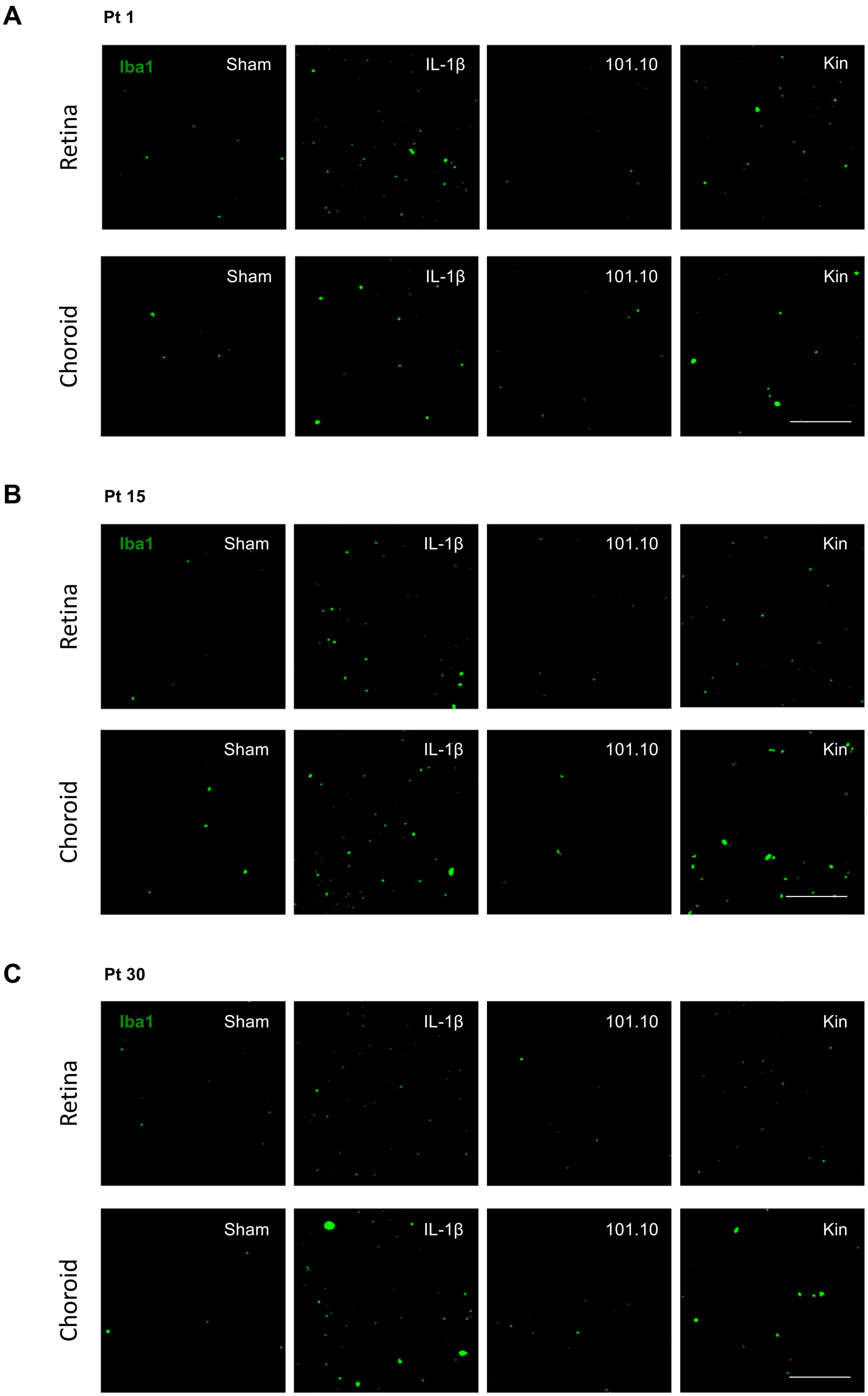

Supplemental figure 3. <u>Chronic post-natal infiltration of iba-1+ cells in the retina and sub-retina.</u> **A-C**, representative images of Iba-1-stained flatmounts of retinas and choroids from pups at Pt 1 (A), Pt 15 (B) and Pt 30 (C) previously exposed to the indicated treatments in utero (Fig. 1); images were taken with confocal microscope. Scale bar, 15 μm.

Supplementary figure 4

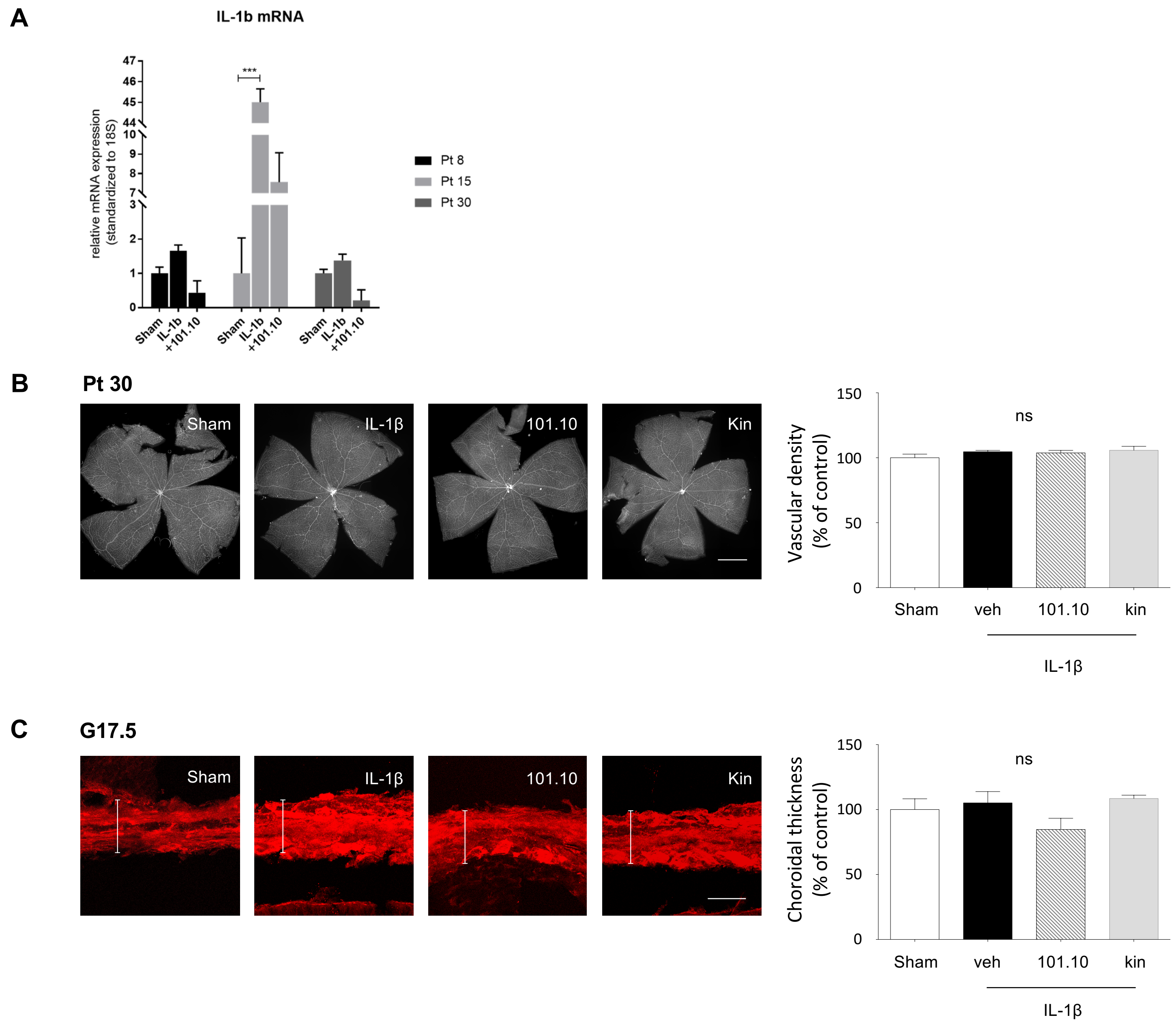

Supplemental Figure 4. <u>Retinal inflammation and vessel density one month after term, and choroid thickness during gestation</u>. **A,** retina IL-1 mRNA expression (relative to 18S) at Pt 8, Pt 15 and Pt 30. Values are presented as mean ± S.E.M. ***$p<0.0001$ by one-way ANOVA with Dunnett's post-analysis; n=3/treatment group. **B**, Lectin-stained flat-mounts of retinas from pups at Pt 30 showing vascularization and vascular density. Images are representative of 4 separate pups per treatment group. Scale bar, 2500 µm. Bottom panel shows quantification of the vascular area, n=4 pups/group. Values are presented as mean ± S.E.M. Data were considered non-significant based on one-way ANOVA with Dunnett's post-analysis. **C**, representative images (top panels) and quantification (bottom panels) of lectin-stained cross-sections of choroids from pups at G17.5 previously exposed to the indicated treatments *in utero*. Vertical bars represent the average choroidal thickness. Scale bar, 28 µm. Bottom panel shows quantification of the vascular area, n=4 pups/group. Values are presented as mean ± S.E.M. Data were considered non-significant based on one-way ANOVA with Dunnett's post-analysis.

Supplementary figure 5

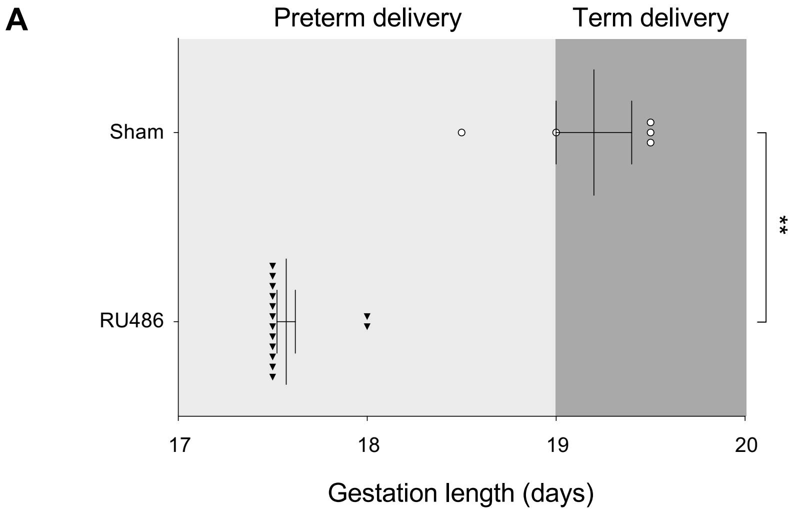

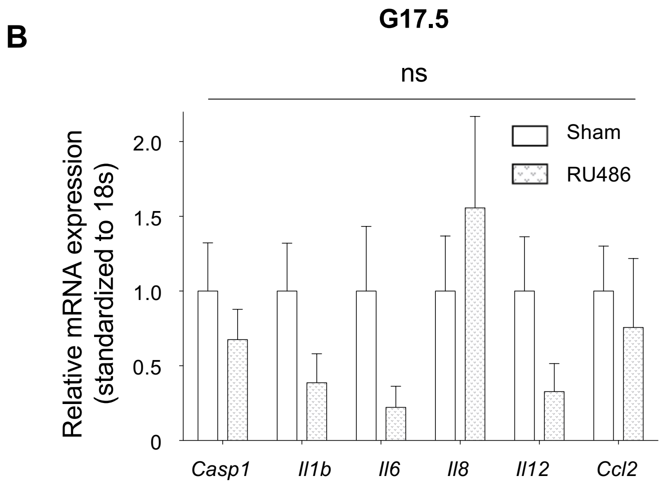

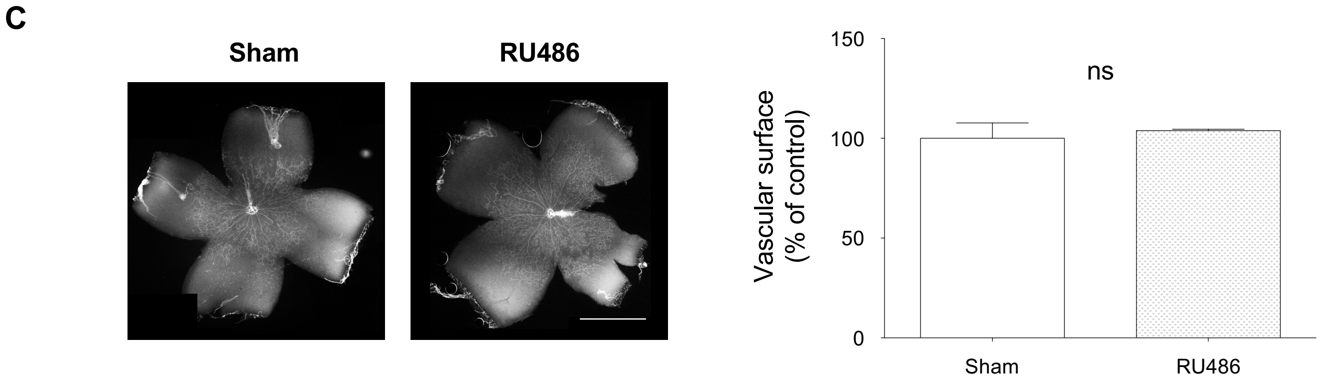

Supplemental Figure 5. <u>Gestation length, ocular inflammation and vascular growth of RU486-induced PTB.</u> **A**, gestation length upon RU486 stimulation. RU486 or vehicle were administered intraperitoneally at G17. n=5-14 dams/group. Values are presented as mean ± S.E.M. **p<0.01 by t-test analysis. **B**, foetal eyes were collected at G17.5 following vehicle or RU486 stimulation to perform quantitative PCR of indicated gene transcripts. Results are relative to 18S and plotted as fold change vs. the control groups. n=3-5 dams/group; 4 foetal eyes per sample. Values are presented as mean ± S.E.M. Data were considered non-significant based on one-way ANOVA with Dunnett's post-analysis. **C**, vascular surface was measured on lectin-stained flat-mounts of retinas from pups at Pt4 previously exposed to vehicle or RU486 *in utero*; n=3-4 pups/group. Values are presented as mean ± S.E.M. Data were considered non-significant (by t-test).

Supplementary figure 6

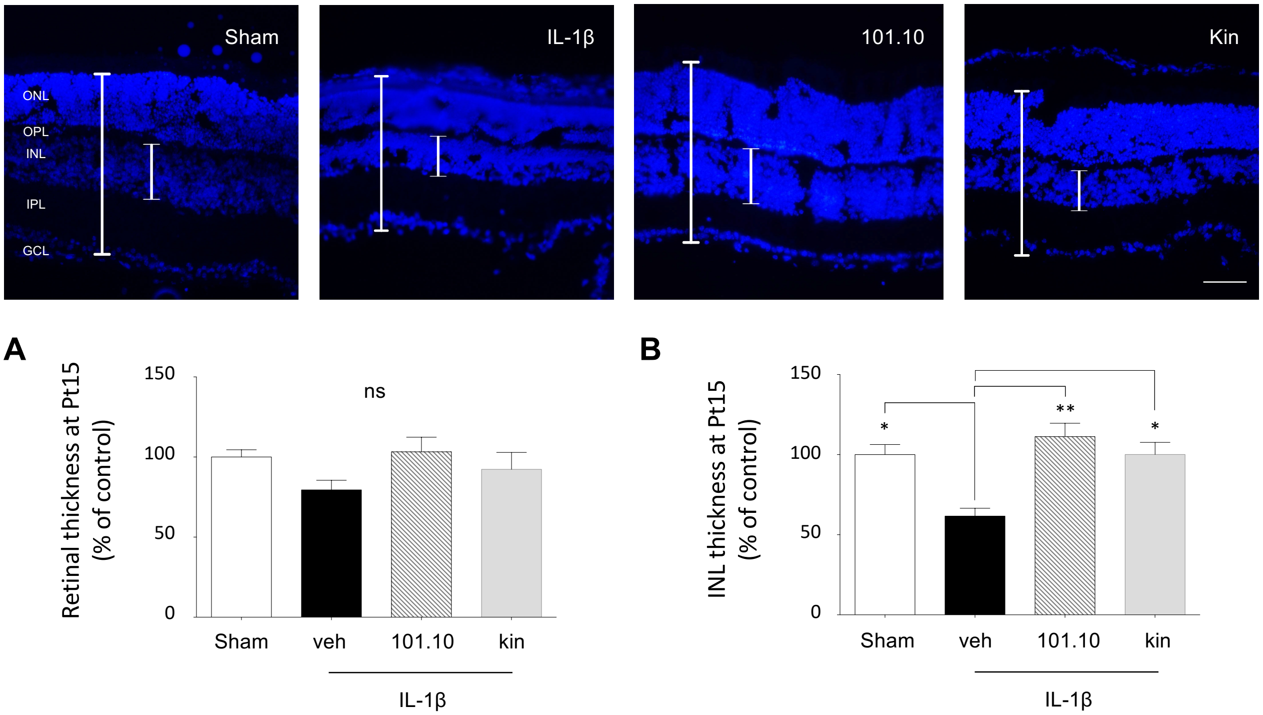

Supplemental Figure 6. Retinal thickness at Pt 15. Top panel: DAPI-stained cross-sections of retina at Pt 15 previously exposed to the indicated treatments *in utero* (Fig. 1). **A, B,** full retina (ONL-GCL) and INL thickness of indicated groups. Values are presented as mean ± S.E.M of n=3-4 pups/group; *p<0.05, **p<0.01 by one-way ANOVA with Dunnett's post-analysis.
